# Supplementary material for: Infection- or AZD1222 vaccine-mediated immunity reduces SARS-CoV-2 transmission but increases Omicron competitiveness in hamsters
Source: Nat Commun. 2023 Oct 18;14:6592. doi: 10.1038/s41467-023-42346-8 (PMC10584863; doi:10.1038/s41467-023-42346-8)
Supplement: Supplementary file 1 — Supplementary Information [file 41467_2023_42346_MOESM1_ESM.pdf]

**Supporting Information for**

# Infection- or AZD1222 vaccine-mediated immunity reduces SARS-CoV-2 transmission but increases Omicron competitiveness in hamsters

Julia R. Port*[1], Claude Kwe Yinda*[1], Jade C. Riopelle[1], Zachary A. Weishampel[1], Taylor A. Saturday[1], Victoria A. Avanzato[1], Jonathan E. Schulz[1], Myndi G. Holbrook[1], Kent Barbian[2], Rose Perry-Gottschalk[3], Elaine Haddock[1], Craig Martens[2], Carl. I. Shaia[4], Teresa Lambe[5$], Sarah C. Gilbert[5], Neeltje van Doremalen#[1], Vincent J. Munster#[§1]

1. Laboratory of Virology, Division of Intramural Research, National Institute of Allergy and Infectious Diseases, National Institutes of Health, Hamilton, MT, USA
2. Genomics Research Section, Research Technologies Branch, Division of Intramural Research, National Institute of Allergy and Infectious Diseases, National Institutes of Health, Hamilton, MT, USA
3. Rocky Mountain Visual and Medical Arts Unit, Research Technologies Branch, Division of Intramural Research, National Institute of Allergy and Infectious Diseases, National Institutes of Health, Hamilton, MT, USA
4. Rocky Mountain Veterinary Branch, Division of Intramural Research, National Institute of Allergy and Infectious Diseases, National Institutes of Health, Hamilton, MT, USA
5. The Jenner Institute, Nuffield Department of Medicine, University of Oxford, Oxford, UK

*These authors contributed equally.
#These authors jointly supervised this work.
§ Corresponding author. vincent.munster@nih.gov
$ New address: Chinese Academy of Medical Science Oxford Institute; Oxford Vaccine Group, Department of Paediatrics, University of Oxford, Oxford, UK

**This PDF file includes:**

Figures S1-3
Tables S1-3
Methods
SI References

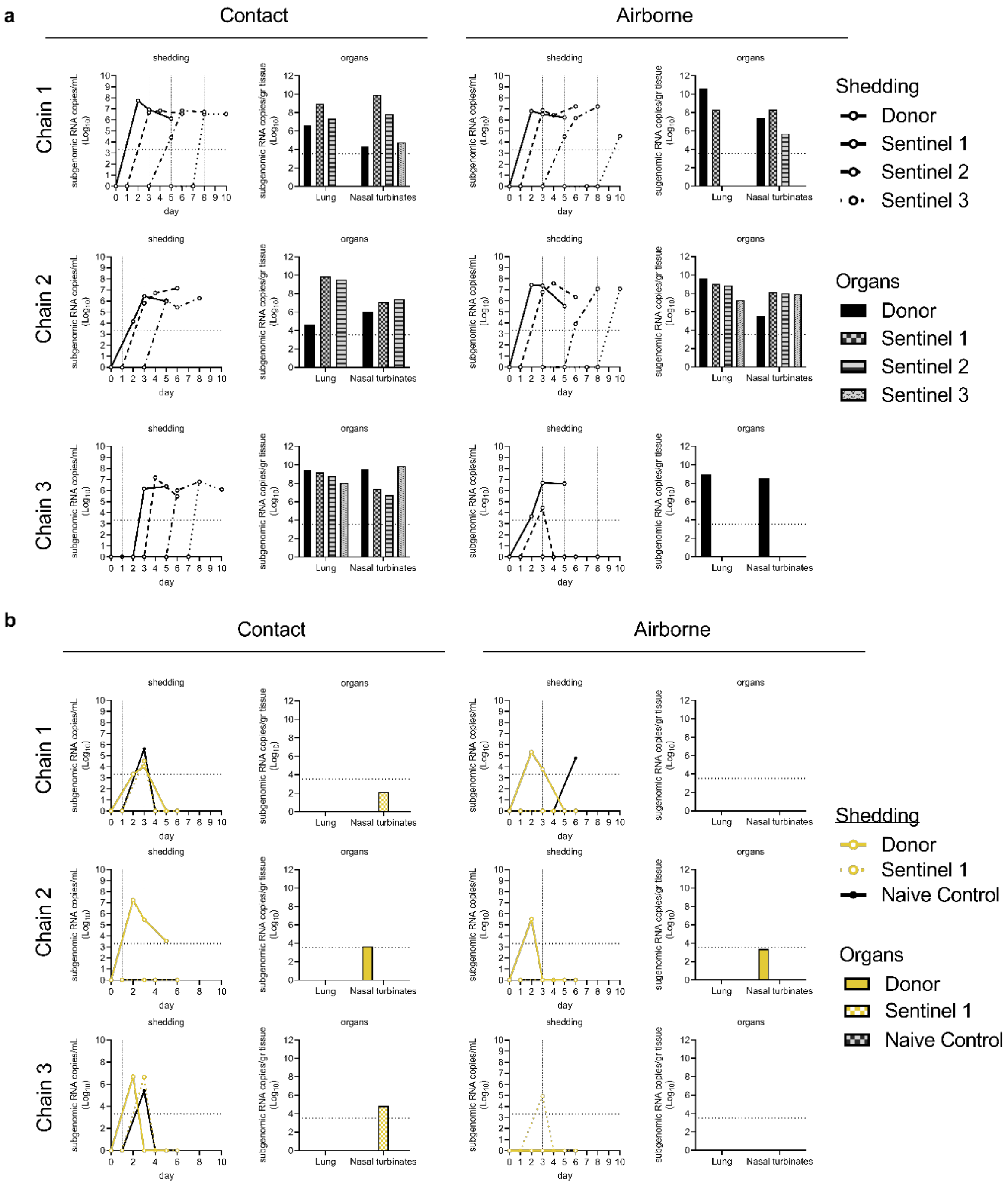
a
Contact
Airborne
Chain 1
Chain 2
Chain 3
shedding
organs
subgenomic RNA copies/mL (Log10)
subgenomic RNA copies/gr tissue (Log10)
day
Lung
Nasal turbinates
Shedding
Donor
Sentinel 1
Sentinel 2
Sentinel 3
Organs
Donor
Sentinel 1
Sentinel 2
Sentinel 3
b
Contact
Airborne
Chain 1
Chain 2
Chain 3
shedding
organs
subgenomic RNA copies/mL (Log10)
subgenomic RNA copies/gr tissue (Log10)
day
Lung
Nasal turbinates
Shedding
Donor
Sentinel 1
Naive Control
Organs
Donor
Sentinel 1
Naive Control

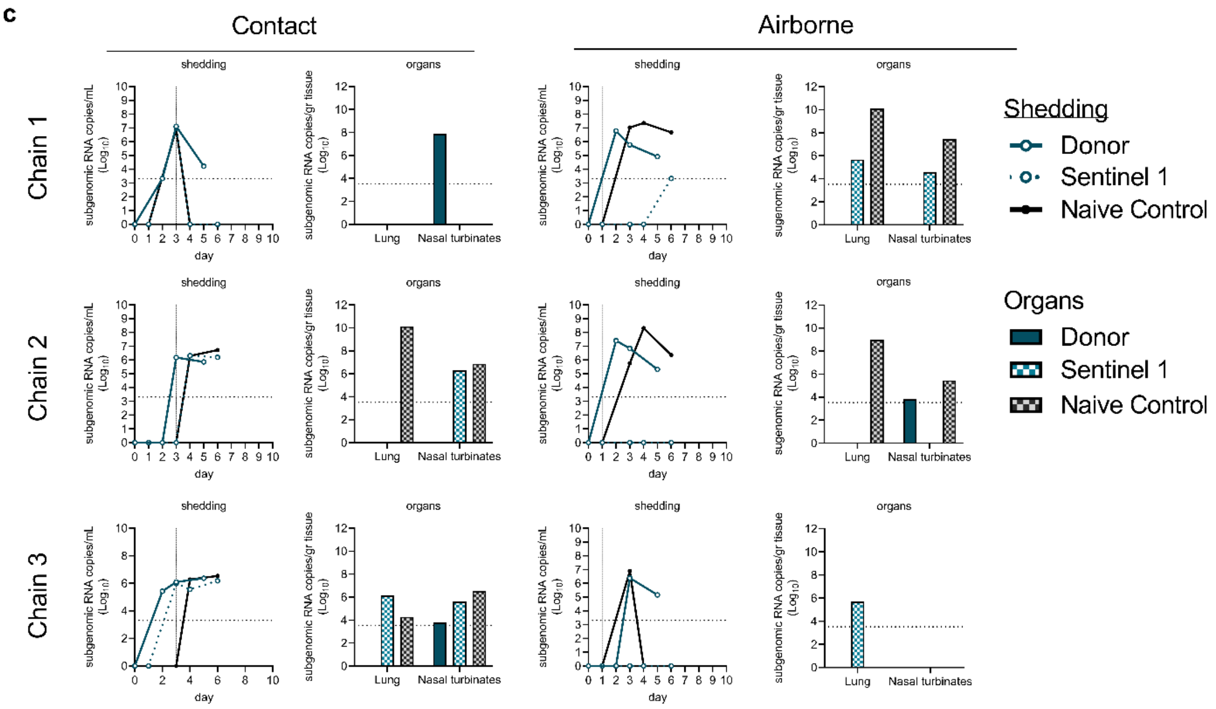


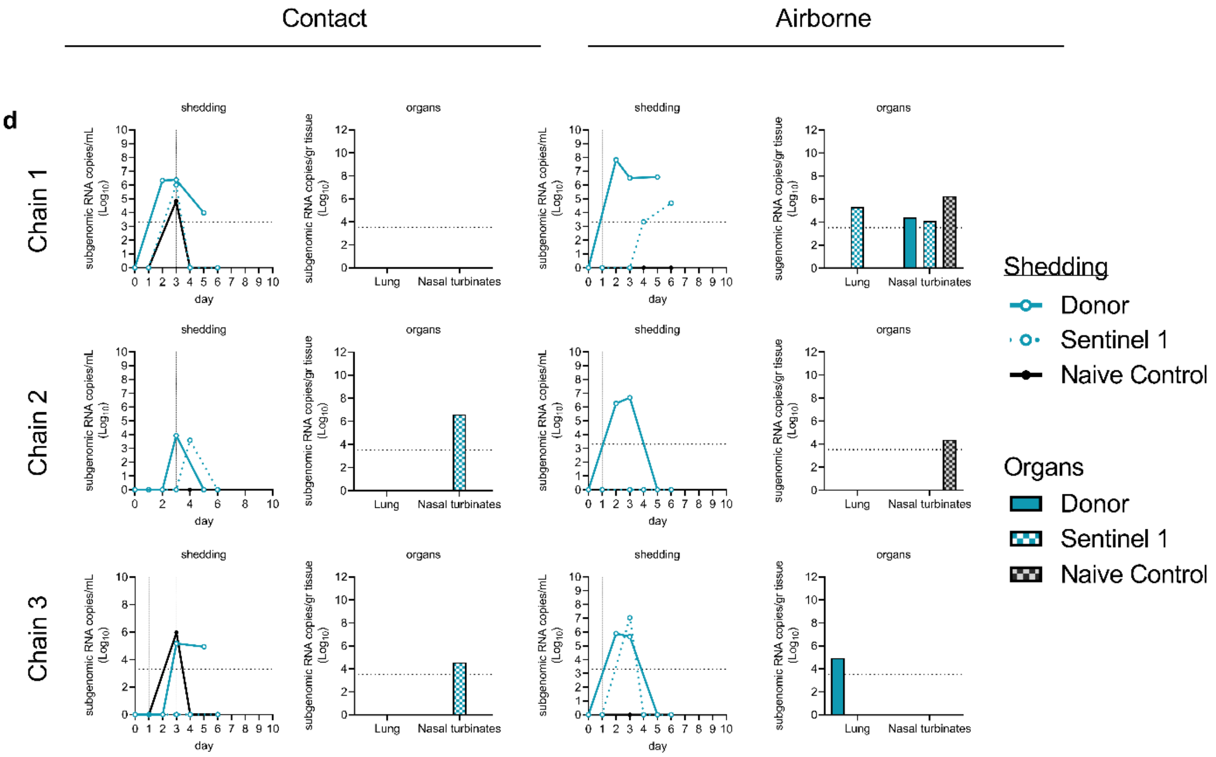


**Fig. S1. Shedding and organ titers of individual animals in each chain.** Animals were grouped into either airborne or contact transmission chains by immune state (N = 3). Chains consisted of either a donor and three consecutive sentinels (naïve chains) or a donor and two

sentinels, one with previous immunity and one naïve control (IM, IN, and PI chains). Donors were infected with 1 x $10^4$ $TCID_{50}$ SARS-CoV-2 at a 1:1 ratio of Omicron and Delta and 24 hours later exposed to either a sentinel and naïve animal (IM, IN, and PI chains) or to the first sentinel (naïve chains) for 48 hours. Naïve chains continued in a similar fashion over four total animals. Replicating virus was measured via oropharyngeal swabs at 2, 3, and 5 DPI/DPE, and in lung and nasal turbinate samples at 5 DPI/DPE. Viral shedding is depicted in oropharyngeal swabs and viral replication in lung and nasal turbinate samples of all animals in contact (left) and airborne (right) chains. Viral shedding and replication are displayed as $log_{10}$ (sgRNA copies/mL) in swabs and $log_{10}$ (sgRNA copies/gram of tissue) in tissues. Horizontal lines indicate limit of detection. Vertical lines indicate exposure events in the chain. A. Naïve chains. B. Previously infected chains, C. Intramuscularly vaccinated chains. D. Intranasally vaccinated chains. Colours refer to legends on the right. Source data are provided as a Source Data file.

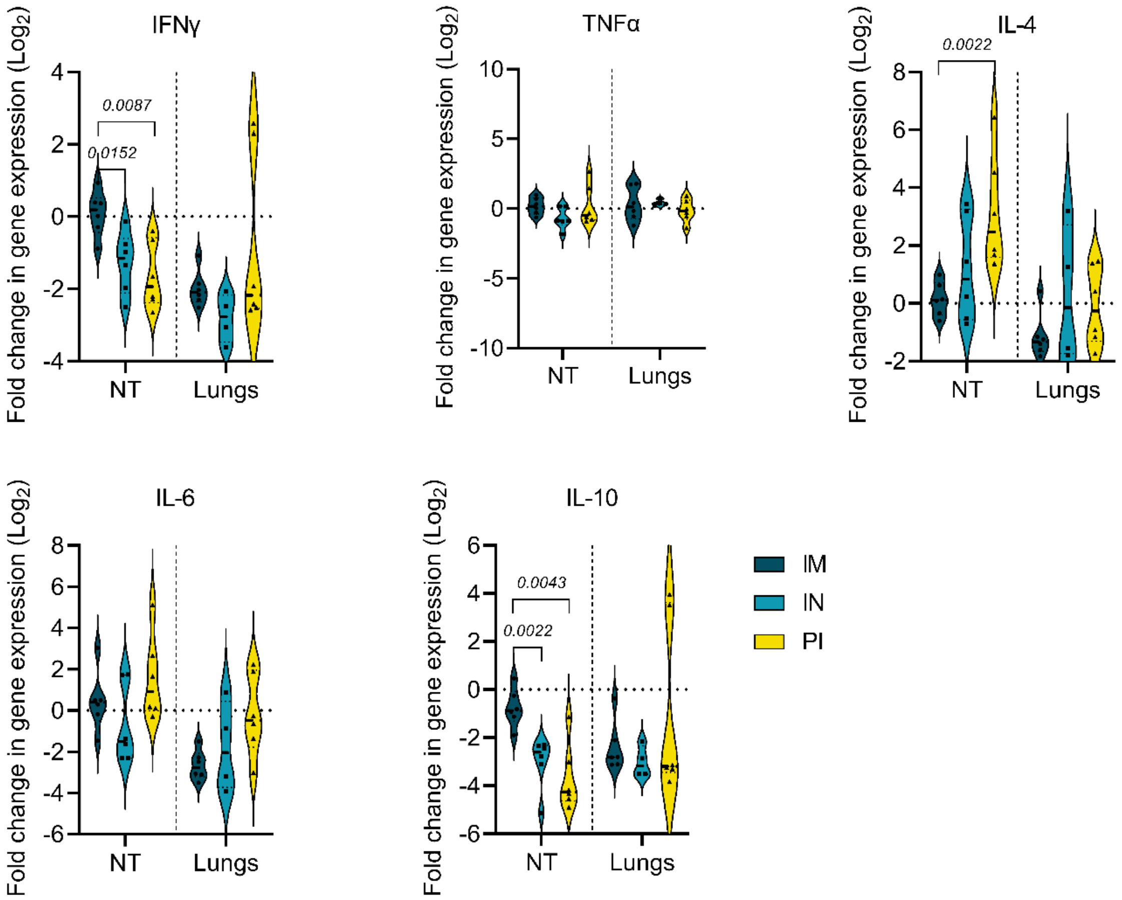


**Fig. S2. Changes in cytokine gene expression post-challenge.** Nasal turbinate (NT) and lung samples were collected at 5 DPI and the fold-change in mRNA expression was calculated for challenged animals with pre-existing immunity over challenged naïve animals. Violin plots depicting median, quantiles, and individual values, N = 6 (lungs IN = 4). Dark blue = IM vaccinated, light blue = IN vaccinated, yellow = PI. Kruskal-Wallis followed by Mann Whitney if statistically significant; p-values stated were significant (<0.05). Source data are provided as a Source Data file.

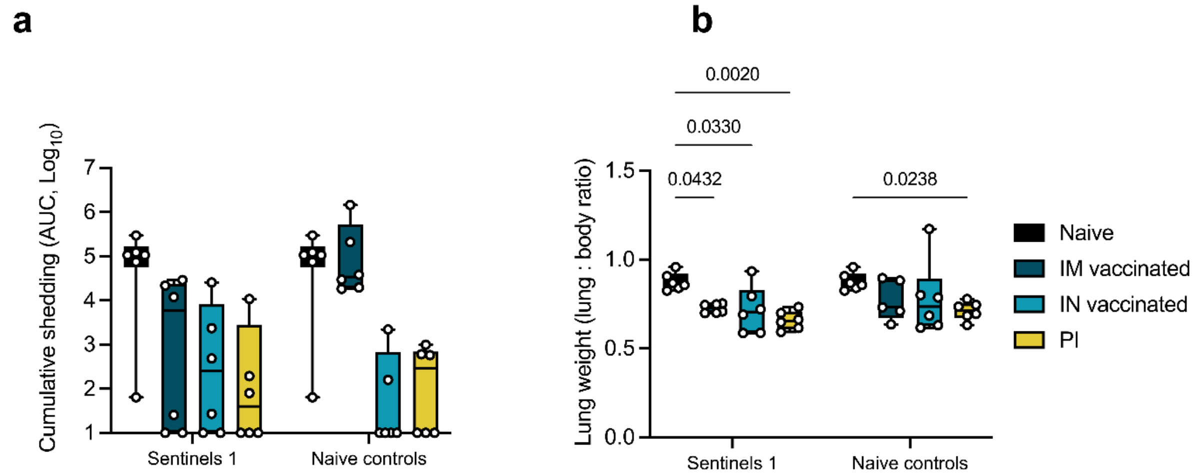


**Fig. S3. Reduction of disease severity and shedding in sentinels.** A. Cumulative shedding. Area under the curve (AUC) of sgRNA measured in oral swabs taken on 2, 3, and 5 DPE (sgRNA copies/rxn/experiment). Whisker-plots depicting median, min and max values, and individual values, N = 6, ordinary two-way ANOVA, followed by Šídák's multiple comparisons test. B. Lung samples were collected at 5 DPE for sentinels and naïve controls. Lung weights (lung:body weight ratio). Whisker-plots depicting median, min and max values, and individual values, N = 6, ordinary two-way ANOVA, followed by Šídák's multiple comparisons test. Black = naïve, dark blue = IM vaccinated, light blue = IN vaccinated, yellow = PI. P-values stated were significant (<0.05). Naïve sentinels 1 are depicted twice in each graph for visualization purposes only. Source data are provided as a Source Data file.

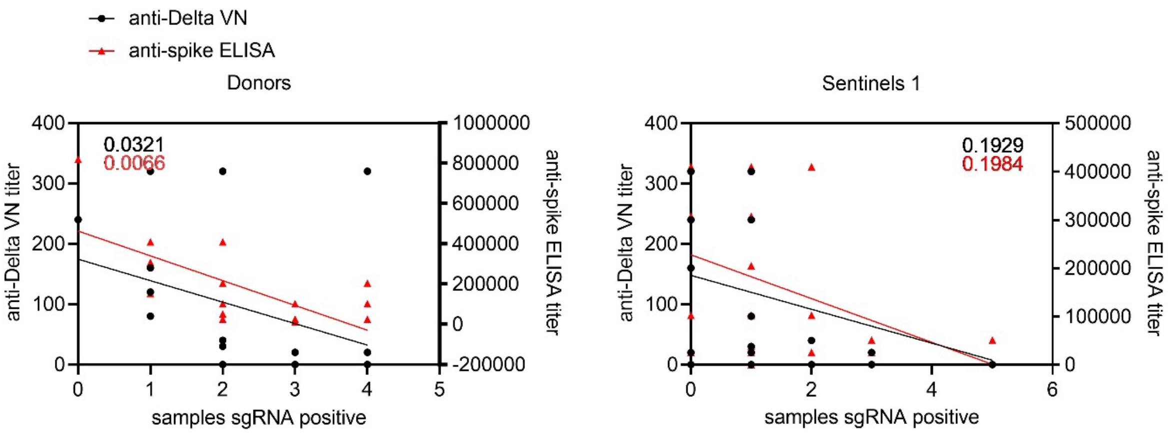


**Fig. S4. Correlation between humoral immune response and protection from infection**. Serum was collected at least 21 days post vaccination against Lineage A or infection with Delta. Correlation between anti-spike IgG response (Lineage A spike), measured by ELISA or individual neutralizing antibody titers against Delta and the amount of positive sgRNA swab or tissue samples (>10 copies/rxn) after challenge (donors) or exposure (sentinels 1). Individuals are depicted, as well as a linear regression line. N = 18, Spearman's correlation, p -values indicated. Source data are provided as a Source Data file.

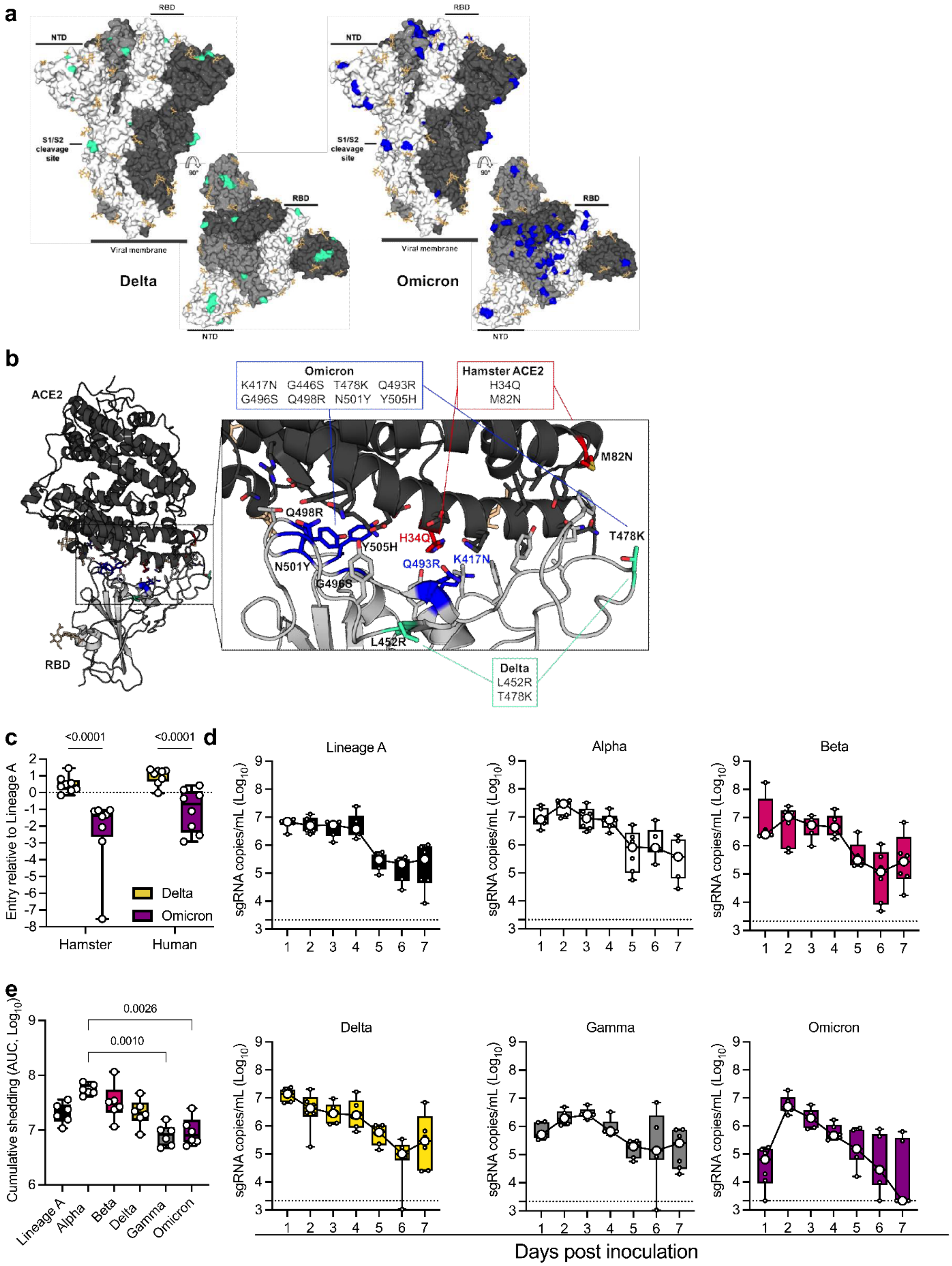


**Fig. S5. Comparison of SARS-CoV-2 variants Omicron and Delta infection in the Syrian hamster.** A. Mutations observed in the SARS-CoV-2 Delta and Omicron VOCs are highlighted

on the structure of SARS-CoV-2 spike (compared to Lineage A, PDB 6ZGE, [1]). The spike trimer is depicted by surface representation with each protomer colored a different shade of gray. The residues at the positions of the spike protein mutations observed in the Delta and Omicron SARS-CoV-2 VOCs are colored teal green (Delta) and blue (Omicron). The receptor binding domain (RBD), N-terminal domain (NTD), and cleavage site are annotated. N-linked glycans are shown as light, orange-colored sticks. B. The structure of the Alpha VOC RBD and human ACE2 complex (PDB 7EKF, [2]) is depicted with cartoon representation. ACE2 is colored dark gray and the RBD is colored light gray. N-linked glycans are shown as light, orange-colored sticks. A box reveals a close-up view of the RBD-ACE2 binding interface. Side chains of the residues participating in the interaction, as identified and described by Lan, et al [3] are shown as sticks. The residues within the RBD that are mutated in the Delta and Omicron VOCs are colored teal green (Delta) and blue (Omicron). Residue T478 is mutated in both Delta and Omicron VOCs but is colored teal green in the figure. Though they do not participate directly in the ACE2 interface, the sidechains of residues L452 and T478 are also shown. The residues that differ between human and hamster ACE2 within the interface are colored red. C. BHK cells expressing either human ACE2 or hamster ACE2 were infected with pseudotyped VSV reporter particles with the spike proteins of Delta or Omicron. Relative entry to a Lineage A control is depicted. Whisker-plots depicting median, min and max values, and individual values, N = 8, ordinary two-way ANOVA, followed by Šídák's multiple comparisons test. D. Viral load as measured by sgRNA in oropharyngeal swabs collected at 1-7 days post intranasal 1,000 $TCID_{50}$ inoculation with Lineage A, Alpha, Beta, Delta, Gamma, or Omicron. Whisker-plots depicting median, min and max values, and individual values, N = 6 (3 males and 3 females). E. Cumulative sgRNA shedding for each variant. Area under the curve for data shown in D (sgRNA copies/ml/experiment). Kruskal-Wallis test. P-values stated were significant (<0.05). Source data are provided as a Source Data file.

**Table S1:** Pathological assessment of IN, IM vaccinated or PI Syrian hamsters on day 5 post challenge. nsf = no significant findings. y = yes. n = no. Nucleoprotein reactivity score: 0 = none, 1 = rare/few, 2 = scattered, 3 = moderate, 4 = numerous, 5 = diffuse.

| Group | Naïve | | | | | | IM vaccinated | | | | | | IN vaccinated | | | | | | Previously infected | | | | | |
|---|---|---|---|---|---|---|---|---|---|---|---|---|---|---|---|---|---|---|---|---|---|---|---|---|
| Lesions visible grossly | y | y | n | y | y | y | y | y | y | ? | y | n | n | n | n | N | n | n | n | n | ? | n | n | n |
| Lung | | | nsf | | | | | | | nsf | | | nsf | nsf | nsf | nsf | | nsf | | nsf | nsf | nsf | nsf | |
| % total area affected | 60 | 60 | | 60 | 70 | 80 | 30 | <10 | 20 | | 20 | <10 | | | | | 10 | | <10 | | | | | <10 |
| Interstitial Pneumonia | | | | | | | | | | | | | | | | | | | | | | | | |
| Syncytial Cell | y | y | n | y | y | y | y | n | n | n | n | n | n | n | n | n | n | n | n | n | n | n | n | n |
| Alveolar inflammation or exudate | y | y | n | y | y | y | y | y | y | n | y | y | n | n | n | n | n | n | n | n | n | n | n | n |
| Endotheleitis, vasculitis | y | y | n | y | y | y | y | y | y | n | y | n | n | n | n | n | n | n | n | n | n | n | n | n |
| Type II pneumocyte hyperplasia | n | n | n | n | n | n | y | n | n | n | n | n | n | n | n | n | n | n | n | n | n | n | n | n |
| Hemorrhage, fibrin and or edema | y | y | n | y | y | y | n | n | y | n | n | n | n | n | n | n | n | n | n | n | n | n | n | n |
| Bronchiolar hyperplasia | y | y | n | y | y | y | y | n | n | n | n | n | n | n | n | n | n | n | n | n | n | n | n | n |
| SARS-CoV-2 IHC bronchioles | 3 | 2 | 0 | 2 | 2 | 2 | 0 | 0 | 3 | 0 | 0 | 1 | 0 | 0 | 0 | 0 | 0 | 0 | 0 | 0 | 0 | 0 | 0 | 0 |
| SARS-CoV-2 IHC alveoli | 4 | 3 | 0 | 3 | 4 | 4 | 1 | 0 | 3 | 0 | 0 | 0 | 0 | 0 | 0 | 0 | 0 | 0 | 0 | 0 | 0 | 0 | 0 | 0 |
| Positive pixel analysis CD3 | 16 | 17 | 7 | 14 | 14 | 16 | 6 | 10 | 10 | 9 | 9 | 10 | 7 | 8 | 8 | 8 | 6 | 9 | 8 | 7 | 8 | 7 | 8 | 6 |
| Positive pixel analysis PAX5 | 3 | 2 | 2 | 2 | 2 | 2 | 1 | 2 | 2 | 2 | 2 | 2 | 2 | 2 | 2 | 2 | 2 | 2 | 1 | 1 | 2 | 2 | 2 | 2 |

**Table S2:** Shedding and tissue titers for each transmission chain (donors and sentinels 1). IM = intramuscularly vaccinated, IN = intranasally vaccinated, PI = Previously infected, BDL = Below qRT-PCR detection limit, sgRNA = sub-genomic RNA. Swab days = 2, 3 and 5. Samples with < 10 sgRNA copies/rxn (approx. > ct = 36) were excluded from sequencing analysis. No data provided if sequencing did not pass quality control or produced no results.

| | | | | sgRNA Ct-value | | | | | % Delta reads | | | | | % Omicron reads | | | | |
|---|---|---|---|---|---|---|---|---|---|---|---|---|---|---|---|---|---|---|
| Transmission route | Chain | Chain link | Immune status | Day 2 swab | Day 3 swab | Day 5 swab | Lung | Nasal turbinates | Day 2 swab | Day 3 swab | Day 5 swab | Lung | Nasal turbinates | Day 2 swab | Day 3 swab | Day 5 swab | Lung | Nasal turbinates |
| air | 1 | Donor | IM | 25 | 28 | 32 | BDL | BDL | 82 | 94 | 95 | | | 17 | 0 | 0 | | |
| air | 1 | Naïve Control | IM | 24 | 23 | 26 | 15 | 23 | 99 | 100 | 100 | 100 | 100 | 0 | 0 | 0 | 0 | 0 |
| air | 1 | Sentinel 1 | IM | BDL | BDL | 37 | 30 | 32 | | | | | 97 | | | | | 0 |
| contact | 1 | Donor | IM | 40 | 24 | 34 | BDL | 22 | | 98 | 98 | | 99 | | 0 | 0 | | 0 |
| contact | 1 | Naïve Control | IM | 25 | BDL | BDL | BDL | BDL | 94 | | | | | 0 | | | | |
| contact | 1 | Sentinel 1 | IM | 24 | BDL | BDL | BDL | BDL | 98 | | | | | 0 | | | | |
| air | 2 | Donor | IM | 23 | 25 | 30 | BDL | 35 | 86 | 92 | 100 | | 100 | 13 | 7 | 0 | | 0 |
| air | 2 | Naïve Control | IM | 28 | 20 | 27 | 19 | 30 | 100 | 99 | 99 | 100 | 100 | 0 | 0 | 0 | 0 | 0 |
| air | 2 | Sentinel 1 | IM | BDL | BDL | BDL | BDL | BDL | | | | | | | | | | |
| contact | 2 | Donor | IM | BDL | 27 | 28 | BDL | BDL | | 99 | 99 | | | | 0 | 0 | | |
| contact | 2 | Naïve Control | IM | BDL | 27 | 25 | 16 | 26 | | 100 | 99 | 100 | 100 | | 0 | 0 | 0 | 0 |
| contact | 2 | Sentinel 1 | IM | BDL | 27 | 27 | BDL | 28 | | 100 | 99 | | 100 | | 0 | 0 | | 0 |
| air | 3 | Donor | IM | BDL | 26 | 31 | BDL | BDL | | 99 | 99 | | | | 0 | 0 | | |
| air | 3 | Naïve Control | IM | 25 | BDL | BDL | BDL | BDL | 99 | | | | | 0 | | | | |
| air | 3 | Sentinel 1 | IM | BDL | BDL | BDL | 28 | BDL | | | | | | | | | | |
| contact | 3 | Donor | IM | 30 | 27 | 27 | BDL | 35 | 94 | 99 | 100 | | | 0 | 0 | 0 | | |
| contact | 3 | Naïve Control | IM | BDL | 27 | 26 | 34 | 31 | | 100 | 100 | | 100 | | 0 | 0 | | 0 |
| contact | 3 | Sentinel 1 | IM | 28 | 29 | 27 | 27 | 29 | 19 | 100 | 99 | 100 | 100 | 80 | 0 | 0 | 0 | 0 |
| air | 1 | Donor | IN | 21 | 26 | 26 | BDL | 33 | 81 | 92 | 98 | | 81 | 18 | 7 | 0 | | 18 |
| air | 1 | Naïve Control | IN | BDL | BDL | BDL | BDL | 27 | | | | | 100 | | | | | 0 |
| air | 1 | Sentinel 1 | IN | BDL | 36 | 32 | 31 | 35 | | | 100 | | 100 | | | 0 | | 0 |

| | | | | | | | | | | | | | | | | | | |
|---|---|---|---|---|---|---|---|---|---|---|---|---|---|---|---|---|---|---|
| contact | 1 | Donor | IN | 27 | 26 | 35 | BDL | BDL | 98 | 95 | 98 | | | 0 | 0 | 0 | | |
| contact | 1 | Naïve Control | IN | 32 | BDL | BDL | BDL | BDL | 100 | | | | | 0 | | | | |
| contact | 1 | Sentinel 1 | IN | 28 | BDL | BDL | BDL | BDL | 100 | | | | | 0 | | | | |
| air | 2 | Donor | IN | 26 | 25 | BDL | BDL | BDL | 84 | 93 | | | | 15 | 0 | | | |
| air | 2 | Naïve Control | IN | BDL | BDL | BDL | BDL | 33 | | | | | 100 | | | | | 0 |
| air | 2 | Sentinel 1 | IN | BDL | BDL | BDL | BDL | BDL | | | | | | | | | | |
| contact | 2 | Donor | IN | BDL | 35 | BDL | BDL | BDL | | 76 | | | | | 23 | | | |
| contact | 2 | Naïve Control | IN | BDL | BDL | BDL | BDL | BDL | | | | | | | | | | |
| contact | 2 | Sentinel 1 | IN | BDL | 36 | BDL | BDL | 27 | | 100 | | | | | 0 | | | |
| air | 3 | Donor | IN | 28 | 29 | BDL | 31 | BDL | 63 | 84 | | | | 36 | 15 | | | |
| air | 3 | Naïve Control | IN | BDL | BDL | BDL | BDL | BDL | | | | | | | | | | |
| air | 3 | Sentinel 1 | IN | 24 | BDL | BDL | BDL | BDL | 75 | | | | | 24 | | | | |
| contact | 3 | Donor | IN | BDL | 31 | 32 | BDL | BDL | | 99 | 100 | | | | 0 | 0 | | |
| contact | 3 | Naïve Control | IN | 28 | BDL | BDL | BDL | BDL | 97 | | | | | 0 | | | | |
| contact | 3 | Sentinel 1 | IN | BDL | BDL | BDL | BDL | 32 | | | | | 100 | | | | | 0 |
| air | 1 | Donor | Naïve | 25 | 26 | 27 | 14 | 23 | 83 | 92 | 99 | 100 | 99 | 16 | 7 | 0 | 0 | 0 |
| air | 1 | Sentinel 1 | Naïve | 24 | 26 | 24 | 20 | 21 | 100 | 90 | 84 | 100 | 99 | 0 | 9 | 15 | 0 | 0 |
| air | 1 | Sentinel 2 | Naïve | 32 | 27 | 24 | BDL | 29 | 100 | 99 | 100 | | 100 | 0 | 0 | 0 | | 0 |
| air | 1 | Sentinel 3 | Naïve | BDL | BDL | 33 | BDL | BDL | | | 100 | | | | | 0 | | |
| contact | 1 | Donor | Naïve | 22 | 25 | 27 | 25 | 33 | 93 | 98 | 98 | 100 | 97 | 0 | 0 | 0 | 0 | 0 |
| contact | 1 | Sentinel 1 | Naïve | 25 | 25 | 26 | 18 | 16 | 96 | 99 | 99 | 99 | 100 | 0 | 0 | 0 | 0 | 0 |
| contact | 1 | Sentinel 2 | Naïve | 33 | 25 | 25 | 23 | 21 | 83 | 100 | 99 | | 100 | 16 | 0 | 0 | | 0 |
| contact | 1 | Sentinel 3 | Naïve | BDL | 26 | 26 | BDL | 32 | | 100 | 100 | | 100 | | 0 | 0 | | 0 |
| air | 2 | Donor | Naïve | 23 | 23 | 30 | 16 | 29 | 82 | 85 | 99 | 99 | 98 | 17 | 14 | 0 | 0 | 0 |
| air | 2 | Sentinel 1 | Naïve | 25 | 22 | 27 | 19 | 22 | 87 | 100 | 100 | 99 | 100 | 12 | 0 | 0 | 0 | 0 |
| air | 2 | Sentinel 2 | Naïve | BDL | 34 | 24 | 20 | 23 | | 100 | 100 | 100 | 100 | | 0 | 0 | 0 | 0 |
| air | 2 | Sentinel 3 | Naïve | BDL | BDL | 24 | 24 | 22 | | | 99 | | 100 | | | 0 | | 0 |
| contact | 2 | Donor | Naïve | 34 | 26 | 28 | 32 | 27 | 100 | 97 | 97 | 100 | 99 | 0 | 0 | 0 | 0 | 0 |

| | | | | | | | | | | | | | | | | | | |
|---|---|---|---|---|---|---|---|---|---|---|---|---|---|---|---|---|---|---|
| contact | 2 | Sentinel 1 | Naïve | 29 | 25 | 24 | 15 | 24 | 100 | 99 | 99 | 100 | 99 | 0 | 0 | 0 | 0 | 0 |
| contact | 2 | Sentinel 2 | Naïve | 28 | 30 | 27 | 17 | 23 | 100 | 100 | 99 | 100 | 100 | 0 | 0 | 0 | 0 | 0 |
| air | 3 | Donor | Naïve | 36 | 25 | 26 | 19 | 20 | 100 | 99 | 99 | 100 | 99 | 0 | 0 | 0 | 0 | 0 |
| air | 3 | Sentinel 1 | Naïve | 33 | BDL | BDL | BDL | BDL | 100 | | | | | 0 | | | | |
| air | 3 | Sentinel 2 | Naïve | BDL | BDL | BDL | BDL | BDL | | | | | | | | | | |
| contact | 3 | Donor | Naïve | BDL | 25 | 28 | 21 | 16 | | 100 | 99 | 100 | 100 | | 0 | 0 | 0 | 0 |
| contact | 3 | Sentinel 1 | Naïve | BDL | 27 | 27 | 17 | 17 | | 99 | 99 | 99 | | | 0 | 0 | 0 | |
| contact | 3 | Sentinel 2 | Naïve | BDL | 24 | 30 | 18 | 25 | | 97 | 99 | 100 | 100 | | 0 | 0 | 0 | 0 |
| contact | 3 | Sentinel 3 | Naïve | BDL | 28 | 25 | 20 | 27 | | 100 | 99 | 100 | 100 | | 0 | 0 | 0 | 0 |
| air | 1 | Donor | PI | 32 | 35 | BDL | BDL | BDL | 0 | 0 | | | | 93 | 95 | | | |
| air | 1 | Naïve Control | PI | BDL | BDL | 32 | BDL | BDL | | | 100 | | | | | 0 | | |
| air | 1 | Sentinel 1 | PI | BDL | BDL | BDL | BDL | BDL | | | | | | | | | | |
| contact | 1 | Donor | PI | 37 | 35 | BDL | BDL | BDL | | 100 | | | | | 0 | | | |
| contact | 1 | Naïve Control | PI | 29 | BDL | BDL | BDL | BDL | | | | | | | | | | |
| contact | 1 | Sentinel 1 | PI | 33 | BDL | BDL | BDL | 39 | 86 | | | | | 13 | | | | |
| air | 2 | Donor | PI | 29 | BDL | BDL | BDL | 37 | 0 | | | | | 95 | | | | |
| air | 2 | Naïve Control | PI | BDL | BDL | BDL | BDL | BDL | | | | | | | | | | |
| air | 2 | Sentinel 1 | PI | BDL | BDL | BDL | BDL | BDL | | | | | | | | | | |
| contact | 2 | Donor | PI | 23 | 30 | 37 | BDL | 35 | 95 | 96 | 57 | | 95 | 0 | 0 | 42 | | 0 |
| contact | 2 | Naïve Control | PI | BDL | BDL | BDL | BDL | BDL | | | | | | | | | | |
| contact | 2 | Sentinel 1 | PI | BDL | BDL | BDL | BDL | BDL | | | | | | | | | | |
| air | 3 | Donor | PI | BDL | BDL | BDL | BDL | BDL | | | | | | | | | | |
| air | 3 | Naïve Control | PI | BDL | BDL | BDL | BDL | BDL | | | | | | | | | | |
| air | 3 | Sentinel 1 | PI | 32 | BDL | BDL | BDL | BDL | 91 | | | | | 8 | | | | |
| contact | 3 | Donor | PI | 25 | BDL | BDL | BDL | BDL | 100 | | | | | 0 | | | | |
| contact | 3 | Naïve Control | PI | 30 | BDL | BDL | BDL | BDL | 100 | | | | | 0 | | | | |
| contact | 3 | Sentinel 1 | PI | 25 | BDL | BDL | BDL | 32 | 100 | | | | | 0 | | | | |

**Table S3:** Humoral immune parameters for each transmission chain (donors, sentinels). IM = intramuscularly vaccinated, IN = intranasally vaccinated, PI = Previously infected, NA = sample not available for analysis.

| | | | | spike ELISA | | virus neutralization | | | | pre-challenge variant-specific antibodies | | | | | | post-challenge variant-specific antibodies | | | | | |
|---|---|---|---|---|---|---|---|---|---|---|---|---|---|---|---|---|---|---|---|---|---|
| Transmission route | Chain | Chain link | Immune status | Pre-challenge titer | Post-challenge titer | Pre-challenge titer against Delta | Pre-challenge titer against Omicron | Post-challenge titer against Delta | Post-challenge titer against Omicron | Lineage A | Alpha | Beta | Delta | Gamma | Omicron | Lineage A | Alpha | Beta | Delta | Gamma | Omicron |
| air | 1 | Naïve Control 2 | IM | NA | NA | NA | NA | NA | NA | NA | NA | NA | NA | NA | NA | 225 | 229 | 201 | 163 | 187.5 | 174 |
| air | 1 | Naïve Control 3 | IM | NA | NA | NA | NA | NA | NA | NA | NA | NA | NA | NA | NA | 204 | 185.5 | 172 | 124.5 | 126.5 | 287 |
| air | 1 | Sentinel 2 | IM | 12800 | 12800 | 0 | 10 | 60 | 10 | 8872 | 7362.5 | 1548.5 | 3906 | 2807.5 | 539.5 | 6475 | 5929 | 2394 | 4833 | 3782 | 682 |
| air | 1 | Sentinel 3 | IM | 25600 | 25600 | 0 | 0 | 30 | 0 | 10275 | 6937 | 1868 | 4635.5 | 6003.5 | 981 | 6338 | 4211 | 1660 | 3990 | 4740 | 813 |
| air | 2 | Naïve Control 2 | IM | NA | NA | NA | NA | NA | NA | NA | NA | NA | NA | NA | NA | 134.5 | 143.5 | 126.5 | 88.5 | 110.5 | 122 |
| air | 2 | Naïve Control 3 | IM | NA | NA | NA | NA | NA | NA | NA | NA | NA | NA | NA | NA | 210 | 209 | 200 | 149 | 154 | 177 |
| air | 2 | Sentinel 2 | IM | 25600 | 25600 | 0 | 0 | 40 | 10 | 14111 | 10683.5 | 3039 | 7358.5 | 3489 | 1064.5 | 9190 | 7314 | 4144 | 7326 | 5374 | 1868 |
| air | 2 | Sentinel 3 | IM | 12800 | 25600 | 0 | 0 | 30 | 0 | 7051 | 5115 | 1437 | 3886.5 | 1873 | 628 | 4003 | 2660 | 1515 | 3005 | 3080 | 629 |
| air | 3 | Donor | IM | 25600 | 204800 | 0 | 0 | 320 | 160 | 15559 | 11207 | 3172.5 | 9197.5 | 3624.5 | 773.5 | 17544 | 14468 | 9299 | 14224 | 9116 | 3577 |
| air | 3 | Naïve Control | IM | NA | NA | NA | NA | NA | NA | NA | NA | NA | NA | NA | NA | 175 | 194.5 | 214 | 143 | 175 | 199.5 |
| air | 3 | Sentinel 1 | IM | 0 | 0 | 0 | 0 | 0 | 0 | 4588 | 3362 | 1151.5 | 1982 | 1673.5 | 469.5 | 1259 | 1088 | 605 | 922 | 759 | 308 |
| air | 1 | Donor | IM | 25600 | 307200 | 0 | 0 | 640 | 240 | 8599.5 | 7160 | 3077 | 5781.5 | 2785 | 904.5 | 10518 | 8734 | 5663 | 8850 | 7336 | 2042 |
| air | 1 | Naïve Control | IM | NA | NA | NA | NA | NA | NA | NA | NA | NA | NA | NA | NA | 276.5 | 257 | 208 | 104 | 179.5 | 185 |
| air | 1 | Sentinel 1 | IM | 25600 | 25600 | 0 | 0 | 80 | 20 | 15357 | 9581.5 | 2884 | 7039.5 | 3514 | 819.5 | 5988 | 3285 | 1802 | 3358 | 3388 | 526 |
| air | 2 | Donor | IM | 25600 | 204800 | 0 | 0 | 960 | 120 | 9971.5 | 6740.5 | 1563.5 | 5578.5 | 2662 | 395 | 26804 | 22179 | 11636 | 22517 | 20067 | 5302 |
| air | 2 | Naïve Control | IM | NA | NA | NA | NA | NA | NA | NA | NA | NA | NA | NA | NA | 201.5 | 203.5 | 225 | 134.5 | 166.5 | 203.5 |
| air | 2 | Sentinel 1 | IM | 25600 | 12800 | 0 | 0 | 80 | 0 | 13238.5 | 9276 | 2246.5 | 6619 | 2369.5 | 572.5 | 6419 | 4567 | 1922 | 4830 | 3073 | 481 |
| contact | 1 | Donor | IM | 25600 | 204800 | 20 | 0 | 240 | 80 | 16743.5 | 13326 | 2819 | 8573.5 | 3601 | 1267.5 | 32519 | 28087 | 17345 | 27140 | 25087 | 8295 |
| contact | 1 | Naïve Control | IM | NA | NA | NA | NA | NA | NA | NA | NA | NA | NA | NA | NA | 184 | 193.5 | 219.5 | 135 | 135.5 | 177 |
| contact | 1 | Sentinel 1 | IM | 25600 | 12800 | 20 | 0 | 20 | 0 | 18251.5 | 13077.5 | 4640 | 9481 | 7430 | 1339.5 | 6065 | 4194 | 3272 | 5522 | 4329 | 1159 |
| contact | 2 | Donor | IM | 25600 | 819200 | 0 | 0 | 1280 | 60 | 8307 | 5499.5 | 1489.5 | 3626.5 | 1513.5 | 345 | 39741 | 29212 | 16450 | 32467 | 22736 | 9082 |
| contact | 2 | Naïve Control | IM | NA | NA | NA | NA | NA | NA | NA | NA | NA | NA | NA | NA | 165.5 | 149.5 | 165 | 102.5 | 194.5 | 127 |
| contact | 2 | Sentinel 1 | IM | 25600 | 51200 | 0 | 0 | 80 | 0 | 9442.5 | 6783.5 | 2007 | 5154 | 2006.5 | 743 | 6829 | 5457 | 3030 | 5879 | 3887 | 1198 |
| contact | 3 | Donor | IM | 12800 | 307200 | 0 | 0 | 320 | 10 | 11302.5 | 6796 | 1577 | 3882.5 | 1811.5 | 492.5 | 24484 | 18982 | 11163 | 21790 | 14532 | 3841 |
| contact | 3 | Naïve Control | IM | NA | NA | NA | NA | NA | NA | NA | NA | NA | NA | NA | NA | 2725.5 | 214.5 | 203 | 140.5 | 165 | 181.5 |
| contact | 3 | Sentinel 1 | IM | 51200 | 76800 | 0 | 0 | 240 | 0 | 24218.5 | 15132.5 | 3701.5 | 13232.5 | 6625 | 1409 | 11510 | 8410 | 4185 | 9750 | 7879 | 1739 |
| air | 1 | Naïve Control 2 | IN | NA | NA | NA | NA | NA | NA | NA | NA | NA | NA | NA | NA | 202 | 192.5 | 152 | 97 | 179 | 142.5 |

| | | | | | | | | | | | | | | | | | | | | | |
|---|---|---|---|---|---|---|---|---|---|---|---|---|---|---|---|---|---|---|---|---|---|
| air | 1 | Naïve Control 3 | IN | NA | NA | NA | NA | NA | NA | NA | NA | NA | NA | NA | NA | 153 | 167.5 | 161 | 115 | 126.5 | 144 |
| air | 1 | Sentinel 2 | IN | 102400 | 25600 | 160 | 0 | 160 | 0 | 18654.5 | 12321.5 | 5946.5 | 14155.5 | 8430 | 901 | 8092 | 5516 | 3554 | 6607 | 6090 | 734 |
| air | 1 | Sentinel 3 | IN | 51200 | 102400 | 20 | 10 | 60 | 60 | 21121.5 | 14226.5 | 4345 | 12966.5 | 6829 | 1715.5 | 15642 | 11446 | 6864 | 10732 | 12066 | 2580 |
| air | 2 | Naïve Control 2 | IN | NA | NA | NA | NA | NA | NA | NA | NA | NA | NA | NA | NA | 195.5 | 188 | 194 | 130.5 | 160.5 | 163.5 |
| air | 2 | Naïve Control 3 | IN | NA | NA | NA | NA | NA | NA | NA | NA | NA | NA | NA | NA | 279.5 | 247.5 | 207.5 | 189 | 269 | 151 |
| air | 2 | Sentinel 2 | IN | 25600 | 102400 | 20 | 0 | 120 | 0 | 13333 | 8894.5 | 2597.5 | 6319.5 | 3660 | 1642.5 | 21341 | 17102 | 8831 | 16033 | 11495 | 5162 |
| air | 2 | Sentinel 3 | IN | 51200 | 204800 | 40 | 30 | 120 | 30 | 35071 | 26897 | 9294 | 18631.5 | 10813 | 3873 | 29222 | 23231 | 14650 | 23339 | 17456 | 7084 |
| air | 3 | Donor | IN | 204800 | 409600 | 30 | 0 | 1280 | 960 | 32627 | 26304 | 4571 | 17189 | 5621 | 2254 | 75480 | 69977 | 45669 | 60304 | 54882 | 25180 |
| air | 3 | Naïve Control | IN | NA | NA | NA | NA | NA | NA | NA | NA | NA | NA | NA | NA | 248 | 228 | 200.5 | 148 | 141.5 | 206 |
| air | 3 | Sentinel 1 | IN | 307200 | 102400 | 30 | 20 | 240 | 80 | 63281.5 | 43021 | 10080 | 30283.5 | 21163.5 | 4397.5 | 25934 | 16953 | 11330 | 17340 | 20516 | 4159 |
| air | 1 | Donor | IN | 102400 | 819200 | 20 | 10 | 2560 | 240 | 17130.5 | 13665 | 4560 | 9842 | 5318 | 2147 | 77893 | 69821 | 47948 | 76692 | 64141 | 20137 |
| air | 1 | Naïve Control | IN | NA | NA | NA | NA | NA | NA | NA | NA | NA | NA | NA | NA | 153 | 183 | 170 | 117 | 119.5 | 163.5 |
| air | 1 | Sentinel 1 | IN | 51200 | 51200 | 20 | 0 | 240 | 0 | 13633.5 | 7299.5 | 2638 | 6143.5 | 10384 | 779.5 | 12721 | 8875 | 4065 | 7528 | 9664 | 1137 |
| air | 2 | Donor | IN | 102400 | 819200 | 30 | 0 | 2560 | 1280 | 18251.5 | 11373.5 | 3365.5 | 10029.5 | 5736 | 992 | 70484 | 56418 | 39148 | 64785 | 59592 | 16958 |
| air | 2 | Naïve Control | IN | NA | NA | NA | NA | NA | NA | NA | NA | NA | NA | NA | NA | 246.5 | 260 | 229.5 | 162 | 235.5 | 201 |
| air | 2 | Sentinel 1 | IN | 102400 | 102400 | 20 | 0 | 120 | 30 | 17617.5 | 14478 | 6240.5 | 11090 | 6330.5 | 1632.5 | 12339 | 10436 | 6460 | 10860 | 6871 | 2041 |
| contact | 1 | Donor | IN | 102400 | 409600 | 20 | 10 | 480 | 1280 | 18493 | 15194.5 | 5024.5 | 10525 | 3922.5 | 1419 | 67260 | 61139 | 46506 | 57046 | 49177 | 25578 |
| contact | 1 | Naïve Control | IN | NA | NA | NA | NA | NA | NA | NA | NA | NA | NA | NA | NA | 691.5 | 624.5 | 394.5 | 382 | 485 | 271 |
| contact | 1 | Sentinel 1 | IN | 102400 | 102400 | 80 | 0 | 240 | 40 | 28134.5 | 22233 | 6303 | 16121 | 8088 | 2434.5 | 14841 | 12908 | 9179 | 11683 | 9760 | 2925 |
| contact | 2 | Donor | IN | 153600 | 819200 | 80 | 40 | 1280 | 960 | 56527.5 | 47228.5 | 14040 | 28777 | 18741 | 7338 | 90648 | 77342 | 60164 | 81921 | 69626 | 30869 |
| contact | 2 | Naïve Control | IN | NA | NA | NA | NA | NA | NA | NA | NA | NA | NA | NA | NA | 169.5 | 182 | 161.5 | 120 | 144.5 | 153.5 |
| contact | 2 | Sentinel 1 | IN | 102400 | 153600 | 40 | 0 | 160 | 30 | 71702.5 | 47943.5 | 14011 | 36254.5 | 21275 | 5457.5 | 35493 | 29902 | 17418 | 29716 | 26537 | 6445 |
| contact | 3 | Donor | IN | 51200 | 102400 | 40 | 0 | 1280 | 640 | 18729.5 | 18111.5 | 3655.5 | 6977.5 | 5931.5 | 1819 | 52008 | 58123 | 40191 | 47389 | 44985 | 13766 |
| contact | 3 | Naïve Control | IN | NA | NA | NA | NA | NA | NA | NA | NA | NA | NA | NA | NA | 309.5 | 445.5 | 235 | 160 | 214.5 | 215.5 |
| contact | 3 | Sentinel 1 | IN | 38400 | 51200 | 20 | 40 | 240 | 160 | 13843.5 | 9101 | 3469.5 | 6311 | 5563 | 1242.5 | 18167 | 14362 | 8812 | 14190 | 10875 | 2652 |
| air | 3 | Donor | Naïve | 0 | NA | NA | NA | NA | NA | NA | NA | NA | NA | NA | NA | 7072 | 5257.5 | 397.5 | 406.5 | 4179 | 386 |
| air | 3 | Sentinel 1 | Naïve | 0 | NA | NA | NA | NA | NA | NA | NA | NA | NA | NA | NA | 580.5 | 1228 | 871 | 600 | 760 | 486.5 |
| air | 3 | Sentinel 2 | Naïve | 0 | NA | NA | NA | NA | NA | NA | NA | NA | NA | NA | NA | 171.5 | 183 | 175.5 | 107.5 | 132.5 | 151.5 |
| air | 1 | Donor | Naïve | 0 | NA | NA | NA | NA | NA | NA | NA | NA | NA | NA | NA | 16474.5 | 8646.5 | 1713.5 | 1862 | 10746 | 1571.5 |
| air | 1 | Sentinel 1 | Naïve | 0 | NA | NA | NA | NA | NA | NA | NA | NA | NA | NA | NA | 441.5 | 464.5 | 492.5 | 255 | 291 | 402 |
| air | 1 | Sentinel 2 | Naïve | 0 | NA | NA | NA | NA | NA | NA | NA | NA | NA | NA | NA | 362.5 | 343 | 246 | 127.5 | 227 | 213.5 |
| air | 1 | Sentinel 3 | Naïve | 0 | NA | NA | NA | NA | NA | NA | NA | NA | NA | NA | NA | 6468 | 5445.5 | 3426 | 4065 | 4973.5 | 1328.5 |
| air | 2 | Donor | Naïve | 0 | NA | NA | NA | NA | NA | NA | NA | NA | NA | NA | NA | 9551.5 | 6714.5 | 747.5 | 866 | 6441 | 1614.5 |

| | | | | | | | | | | | | | | | | | | | | | |
|---|---|---|---|---|---|---|---|---|---|---|---|---|---|---|---|---|---|---|---|---|---|
| air | 2 | Sentinel 1 | Naïve | 0 | NA | NA | NA | NA | NA | NA | NA | NA | NA | NA | NA | 219.5 | 196.5 | 180.5 | 112 | 143 | 165.5 |
| air | 2 | Sentinel 2 | Naïve | 0 | NA | NA | NA | NA | NA | NA | NA | NA | NA | NA | NA | 211.5 | 204.5 | 217 | 131 | 145.5 | 184 |
| air | 2 | Sentinel 3 | Naïve | 0 | NA | NA | NA | NA | NA | NA | NA | NA | NA | NA | NA | 168 | 178.5 | 184.5 | 133.5 | 133.5 | 150.5 |
| contact | 1 | Donor | Naïve | 0 | NA | NA | NA | NA | NA | NA | NA | NA | NA | NA | NA | 8912.5 | 6554.5 | 690.5 | 518 | 5060.5 | 454 |
| contact | 1 | Sentinel 1 | Naïve | 0 | NA | NA | NA | NA | NA | NA | NA | NA | NA | NA | NA | 1259 | 1026 | 652 | 650 | 940 | 389 |
| contact | 1 | Sentinel 2 | Naïve | 0 | NA | NA | NA | NA | NA | NA | NA | NA | NA | NA | NA | 201 | 213 | 217.5 | 127.5 | 135.5 | 198.5 |
| contact | 1 | Sentinel 3 | Naïve | 0 | NA | NA | NA | NA | NA | NA | NA | NA | NA | NA | NA | 345.5 | 334 | 219 | 227 | 243 | 184 |
| contact | 2 | Donor | Naïve | NA | NA | NA | NA | NA | NA | NA | NA | NA | NA | NA | NA | 14381.5 | 7911.5 | 1257.5 | 1170.5 | 10736 | 539 |
| contact | 2 | Sentinel 1 | Naïve | NA | NA | NA | NA | NA | NA | NA | NA | NA | NA | NA | NA | 160.5 | 139 | 141 | 128 | 115.5 | 131.5 |
| contact | 2 | Sentinel 2 | Naïve | NA | NA | NA | NA | NA | NA | NA | NA | NA | NA | NA | NA | 202 | 234 | 215 | 133.5 | 155 | 198 |
| contact | 3 | Donor | Naïve | 0 | NA | NA | NA | NA | NA | NA | NA | NA | NA | NA | NA | 641 | 546.5 | 411 | 363 | 513 | 296.5 |
| contact | 3 | Sentinel 1 | Naïve | NA | NA | NA | NA | NA | NA | NA | NA | NA | NA | NA | NA | 18064 | 12168.5 | 1101.5 | 1350.5 | 11167.5 | 718.5 |
| contact | 3 | Sentinel 2 | Naïve | NA | NA | NA | NA | NA | NA | NA | NA | NA | NA | NA | NA | 225.5 | 233.5 | 227 | 164.5 | 179.5 | 211.5 |
| contact | 3 | Sentinel 3 | Naïve | NA | NA | NA | NA | NA | NA | NA | NA | NA | NA | NA | NA | 232.5 | 238.5 | 200.5 | 159.5 | 188.5 | 205 |
| air | 1 | Naïve Control 2 | PI | NA | NA | NA | NA | NA | NA | NA | NA | NA | NA | NA | NA | 240 | 298 | 251.5 | 177.5 | 180.5 | 252 |
| air | 1 | Naïve Control 3 | PI | NA | NA | NA | NA | NA | NA | NA | NA | NA | NA | NA | NA | 183 | 154.5 | 168.5 | 108 | 139 | 169 |
| air | 1 | Sentinel 2 | PI | 102400 | 307200 | 320 | 0 | 1280 | 320 | 77091.5 | 64443.5 | 27405.5 | 79600 | 25469.5 | 16203.5 | 47334.5 | 44303 | 28614 | 63907 | 26894.5 | 11696.5 |
| air | 1 | Sentinel 3 | PI | 204800 | 204800 | 320 | 0 | 640 | 30 | 66655.5 | 48554.5 | 19968.5 | 69392.5 | 24370 | 8664.5 | 35060 | 31041.5 | 21082 | 45068 | 19088 | 6117.5 |
| air | 2 | Naïve Control 2 | PI | NA | NA | NA | NA | NA | NA | NA | NA | NA | NA | NA | NA | 231 | 252 | 229.5 | 144.5 | 178 | 221 |
| air | 2 | Naïve Control 3 | PI | NA | NA | NA | NA | NA | NA | NA | NA | NA | NA | NA | NA | 185.5 | 180 | 177.5 | 178.5 | 146.5 | 165.5 |
| air | 2 | Sentinel 2 | PI | 102400 | 153600 | 320 | 60 | 960 | 80 | 67804.5 | 54699 | 24231 | 82264.5 | 31855 | 8200 | 35169 | 31507 | 20163 | 54281.5 | 18901.5 | 7387.5 |
| air | 2 | Sentinel 3 | PI | 409600 | 409600 | 160 | 30 | 960 | 160 | 75900.5 | 64294.5 | 25405.5 | 75421.5 | 38502.5 | 15369.5 | 36773.5 | 34585.5 | 27677 | 60129.5 | 28448.5 | 10180 |
| air | 3 | Donor | PI | 819200 | 819200 | 240 | 30 | 2560 | 640 | 97759.5 | 83310.5 | 35128 | 101515.5 | 50381 | 28233 | 52638 | 51036.5 | 42596 | 75964 | 37616 | 19598.5 |
| air | 3 | Naïve Control | PI | NA | NA | NA | NA | NA | NA | NA | NA | NA | NA | NA | NA | 221 | 231.5 | 212 | 138 | 175.5 | 207 |
| air | 3 | Sentinel 1 | PI | 204800 | 204800 | 240 | 40 | 1920 | 960 | 53198.5 | 38329.5 | 13905.5 | 79721.5 | 15449.5 | 7838.5 | 19330.5 | 17545.5 | 14728 | 28954.5 | 11520 | 5096 |
| air | 1 | Donor | PI | 409600 | 204800 | 320 | 240 | 1920 | 1920 | 123928.5 | 110427 | 50020.5 | 132427.5 | 68374 | 26803 | 74852 | 68762.5 | 53904 | 87935 | 57204 | 24813 |
| air | 1 | Naïve Control | PI | NA | NA | NA | NA | NA | NA | NA | NA | NA | NA | NA | NA | 159.5 | 164 | 153 | 79 | 120 | 166.5 |
| air | 1 | Sentinel 1 | PI | 409600 | 102400 | 240 | 0 | 320 | 60 | 81121.5 | 66302 | 27641.5 | 88361 | 30078 | 17041 | 29559 | 26161.5 | 16480.5 | 45111 | 16073.5 | 6515 |
| air | 2 | Donor | PI | 307200 | 409600 | 160 | 0 | 2560 | 1280 | 71450 | 55514 | 21661 | 96791 | 28806.5 | 13905 | 80417 | 76805.5 | 52033 | 112139.5 | 52535.5 | 27554 |
| air | 2 | Naïve Control | PI | NA | NA | NA | NA | NA | NA | NA | NA | NA | NA | NA | NA | 283 | 356 | 317 | 193.5 | 234.5 | 281.5 |
| air | 2 | Sentinel 1 | PI | 307200 | 102400 | 320 | 10 | 960 | 60 | 75529 | 53828 | 21013 | 73745.5 | 23580.5 | 9521 | 38821 | 32743 | 25025 | 53059.5 | 22210.5 | 8108.5 |
| contact | 1 | Donor | PI | 409600 | 614400 | 320 | 20 | 2560 | 480 | 91773 | 82524 | 35151.5 | 105404.5 | 40463 | 14481.5 | 70536 | 66229 | 45650 | 97128 | 45424 | 19512 |

| | | | | | | | | | | | | | | | | | | | | | |
|---|---|---|---|---|---|---|---|---|---|---|---|---|---|---|---|---|---|---|---|---|---|
| contact | 1 | Naïve Control | PI | NA | NA | NA | NA | NA | NA | NA | NA | NA | NA | NA | NA | 173 | 215.5 | 161.5 | 119.5 | 135 | 178 |
| contact | 1 | Sentinel 1 | PI | 409600 | 204800 | 320 | 80 | 1280 | 240 | 103973.5 | 82091.5 | 36887 | 91762.5 | 42246 | 23805 | 45534 | 40183 | 29798.5 | 55114 | 28030 | 14086.5 |
| contact | 2 | Donor | PI | 204800 | 307200 | 320 | 160 | 1280 | 960 | 83254 | 67851.5 | 22721 | 87493 | 29854 | 13598.5 | 71475 | 64482.5 | 47832.5 | 84450.5 | 43852.5 | 22673.5 |
| contact | 2 | Naïve Control | PI | NA | NA | NA | NA | NA | NA | NA | NA | NA | NA | NA | NA | 614 | 557.5 | 436 | 399 | 709.5 | 268 |
| contact | 2 | Sentinel 1 | PI | 409600 | 307200 | 160 | 0 | 640 | 160 | 84023 | 66186.5 | 29457.5 | 102057.5 | 34172 | 13539 | 44630.5 | 38414.5 | 29174.5 | 61470 | 26151 | 13332 |
| contact | 3 | Donor | PI | 409600 | 409600 | 120 | 30 | 1280 | 480 | 82590 | 68282.5 | 29771 | 83558.5 | 42848.5 | 20544.5 | 93009 | 82432 | 61849.5 | 114292 | 62931 | 31692 |
| contact | 3 | Naïve Control | PI | NA | NA | NA | NA | NA | NA | NA | NA | NA | NA | NA | NA | 155.5 | 148.5 | 170.5 | 99 | 120 | 161 |
| contact | 3 | Sentinel 1 | PI | 409600 | 409600 | 480 | 120 | 1280 | 320 | 134347 | 115621.5 | 54286.5 | 139190 | 69516.5 | 26920.5 | 71184.5 | 61945.5 | 46613 | 85393 | 43008.5 | 17968 |

**Table S4:** SARS-CoV-2 isolates used in this study.

| Virus | WHO Description | PANGO Lineage | GISAID$/Genbank Acc* |
|---|---|---|---|
| SARS-CoV-2/human/USA/WA-CDC-WA1/2020 | Lineage A | WA1 | MN985325 |
| England/204820464/2020 | Alpha | B.1.1.7 | EPI_ISL_683466 |
| USA/MD-HP01542/2021 | Beta | B.1.351 | EPI_ISL_890360 |
| hCoV-19/USA/MD-HP03867/2021 | Gamma | P.1 | EPI_ISL_1468644 |
| hCoV-19/USA/KY-CDC-2-4242084/2021 | Delta | B.1.617.2 | EPI_ISL_1823618 |
| hCoV-19/USA/GA-EHC-2811C/2021 | Omicron | B.1.1.529 | EPI_ISL_7171744 |

$GISAID: https://www.epicov.org/epi3/frontend#57217d
*GenBank: https://www.ncbi.nlm.nih.gov/nuccore/MN985325

**Table S5:** *Cytokine gene expression primers*

| Primer name | Sequence (5’ -> 3’) |
|---|---|
| IFNy-F | TTGTTGCTCTGCCTCACTC |
| IFNy-R | CCCTCCATTCACGACATCTAAG |
| IFNy-P | FAM-TACTGCCAGGGCACACTCATTGAA |
| IL10-F | AGCGCTGTCATCGATTTCTC |
| IL10-R | CGCCTTTCTCTTGGAGCTTAT |
| IL10-P | FAM-AAGGCTGTGGAACAGGTGAAGGAT |
| IL4-F | CCACGGAGAAAGACCTCATCTG |
| IL4-R | GGGTCACCTCATGTTGGAAATAAA |
| IL4-P | FAM-CAGGGCTTCCCAGGTGCTTCGCAAGT |
| IL6-F | CCACCAGGAACGAAAGACAA |
| IL6-R | CAGCAGTCCCAAGAAGACAA |
| IL6-P | FAM-AACTTCATAGCTGTTCCTGGAGGGC |
| TNFa-F | GGAGTGGCTGAGCCATCGT |
| TNFa-R | AGCTGGTTGTCTTTGAGAGACATG |
| TNFa-P | FAM-CCAATGCCCTCCTGGCCAACG |

## Methods

*VOC virus shedding comparison*

For the comparison of virus shedding of all VOCs, four-to-six-week-old Syrian golden hamsters (N = 6 per group, Envigo Indianapolis) were inoculated intranasally with 40 µL containing 1 ×

$10^3$ $TCID_{50}$ virus in sterile DMEM. Oropharyngeal swabs were collected in 1 mL of DMEM2 on day post infection 1-7.

*Pseudotype entry assay*

The spike coding sequences for SARS-CoV-2 variant Lineage A, Delta, and Omicron (MN985325, EPI_ISL_2441471, EPI_ISL_6699767, respectively) were truncated by deleting 19 aa at the C-terminus. The spike (S) proteins with the 19 aa deletions of coronaviruses were previously reported to show increased efficiency regarding incorporation into virions of VSV [4, 5]. These sequences were codon optimized for human cells, then appended with a 5′ kozak expression sequence (GCCACC) and 3′ tetra-glycine linker followed by nucleotides encoding a FLAG-tag sequence (DYKDDDDK). These spike sequences were synthesized and cloned into pcDNA3.1$^+$(GenScript). Human ACE2 (Q9BYF1.2, https://www.ncbi.nlm.nih.gov/protein/Q9BYF1.2) and hamster ACE2 (GQ262794.1 https://www.ncbi.nlm.nih.gov/nuccore/GQ262794.1) were synthesized and cloned into pcDNA3.1$^+$ (GenScript). All DNA constructs were verified by Sanger sequencing (ACGT). BHK cells (kindly provided by Marshal Bloom, also available from ATCC {CCL-10, https://www.atcc.org/products/ccl-10)) were seeded in black 96-well plates and transfected the next day with 100 ng plasmid DNA encoding human or hamster ACE2, using polyethylenimine (Polysciences). All downstream experiments were performed 24 h post-transfection. Pseudotype production was carried as previously described [6]. Briefly, plates pre-coated with poly-L-lysine (Sigma–Aldrich) were seeded with 293T cells (kindly provided by Sonja Best, also available from ATCC (CRL-3216 https://www.atcc.org/products/crl-3216)) and transfected the following day with 1,200 ng of empty plasmid and 400 ng of plasmid encoding coronavirus spike or no-spike plasmid control (green fluorescent protein (GFP)). After 24 h, transfected cells were

infected with VSVΔG seed particles pseudotyped with VSV-G, as previously described [6, 7]. After one hour of incubating with intermittent shaking at 37 °C, cells were washed four times and incubated in 2 mL DMEM supplemented with 2% FBS, penicillin/streptomycin and L-glutamine for 48 h. Supernatants were collected, centrifuged at 500 x g for 5 min, aliquoted, and stored at −80 °C. BHK cells previously transfected with ACE2 plasmid of interest were inoculated with equivalent volumes of pseudotype stocks. Plates were then centrifuged at 1200 x g at 4 °C for one hour and incubated overnight at 37 °C. Approximately 18–20 h post-infection, Bright-Glo luciferase reagent (Promega) was added to each well, 1:1, and luciferase was measured. Relative entry was calculated normalizing the relative light unit for spike pseudotypes to the plate relative light unit average for the no-spike control. Each figure shows the data for two technical replicates.

*Structural interaction analysis*

The locations of the described spike mutations in the Delta and Omicron VOCs were highlighted on the SARS-CoV-2 spike structure (PDB 6ZGE [1]). To visualize the molecular interactions at the RBD – ACE2 binding interface, the crystal structure of the Alpha variant RBD and human ACE2 complex (PDB 7EKF [2]) was utilized. All figures were generated using The PyMOL Molecular Graphics System version 2.5.4 (https://www.schrodinger.com/pymol).

## References

1. Wrobel AG*, et al.* SARS-CoV-2 and bat RaTG13 spike glycoprotein structures inform on virus evolution and furin-cleavage effects. *Nature Structural & Molecular Biology* **27**, 763-767 (2020).

2. Han P*, et al.* Molecular insights into receptor binding of recent emerging SARS-CoV-2 variants. *Nature Communications* **12**, 6103 (2021).

3. Lan J*, et al.* Structure of the SARS-CoV-2 spike receptor-binding domain bound to the ACE2 receptor. *Nature* **581**, 215-220 (2020).

4. Fukushi S*, et al.* Vesicular stomatitis virus pseudotyped with severe acute respiratory syndrome coronavirus spike protein. *Journal of General Virology* **86**, 2269-2274 (2005).

5. Kawase M, Shirato K, Matsuyama S, Taguchi F. Protease-Mediated Entry via the Endosome of Human Coronavirus 229E. *Journal of Virology* **83**, 712-721 (2009).

6. Letko M, Marzi A, Munster V. Functional assessment of cell entry and receptor usage for SARS-CoV-2 and other lineage B betacoronaviruses. *Nature Microbiology* **5**, 562-569 (2020).

7. Takada A*, et al.* A system for functional analysis of Ebola virus glycoprotein. *Proceedings of the National Academy of Sciences* **94**, 14764-14769 (1997).
