# Supplementary material for: Infection- or AZD1222 vaccine-mediated immunity reduces SARS-CoV-2 transmission but increases Omicron competitiveness in hamsters
Source: Nat Commun. 2023 Oct 18;14:6592. doi: 10.1038/s41467-023-42346-8 (PMC10584863; doi:10.1038/s41467-023-42346-8)
Supplement: Supplementary file 3 — Reporting Summary [file 41467_2023_42346_MOESM3_ESM.pdf]

nature portfolio

Corresponding author(s): Vincent Munster

Last updated by author(s): 9/1/2023

# Reporting Summary

Nature Portfolio wishes to improve the reproducibility of the work that we publish. This form provides structure for consistency and transparency in reporting. For further information on Nature Portfolio policies, see our Editorial Policies and the Editorial Policy Checklist.

## Statistics

For all statistical analyses, confirm that the following items are present in the figure legend, table legend, main text, or Methods section.

| n/a | Confirmed | |
|---|---|---|
| [ ] | [x] | The exact sample size (*n*) for each experimental group/condition, given as a discrete number and unit of measurement |
| [ ] | [x] | A statement on whether measurements were taken from distinct samples or whether the same sample was measured repeatedly |
| [ ] | [x] | The statistical test(s) used AND whether they are one- or two-sided<br>*Only common tests should be described solely by name; describe more complex techniques in the Methods section.* |
| [ ] | [x] | A description of all covariates tested |
| [ ] | [x] | A description of any assumptions or corrections, such as tests of normality and adjustment for multiple comparisons |
| [ ] | [x] | A full description of the statistical parameters including central tendency (e.g. means) or other basic estimates (e.g. regression coefficient) AND variation (e.g. standard deviation) or associated estimates of uncertainty (e.g. confidence intervals) |
| [ ] | [x] | For null hypothesis testing, the test statistic (e.g. *F*, *t*, *r*) with confidence intervals, effect sizes, degrees of freedom and *P* value noted<br>*Give P values as exact values whenever suitable.* |
| [x] | [ ] | For Bayesian analysis, information on the choice of priors and Markov chain Monte Carlo settings |
| [x] | [ ] | For hierarchical and complex designs, identification of the appropriate level for tests and full reporting of outcomes |
| [x] | [ ] | Estimates of effect sizes (e.g. Cohen's *d*, Pearson's *r*), indicating how they were calculated |

*Our web collection on statistics for biologists contains articles on many of the points above.*

## Software and code

Policy information about availability of computer code

| | |
|---|---|
| Data collection | QuantStudio Software 2.3, ImageScope Positive Pixel Count algorithm (version 9.1), MSD Discovery Workbench Application |
| Data analysis | GraphPad Prism 9, Pymol Molecular Graphics System 2.5.4, FASTX-Toolkit, Bowtie2 version 2.2.928, picard MarkDuplicates, GATk HaplotypeCaller version 4.1.2.029, AdapterRemoval 2.2.2, SAMtools 1.10, ImageScope Positive Pixel Count algorithm 9.1, bcftools 1.9 |

For manuscripts utilizing custom algorithms or software that are central to the research but not yet described in published literature, software must be made available to editors and reviewers. We strongly encourage code deposition in a community repository (e.g. GitHub). See the Nature Portfolio guidelines for submitting code & software for further information.

## Data

Policy information about availability of data

All manuscripts must include a data availability statement. This statement should provide the following information, where applicable:

- Accession codes, unique identifiers, or web links for publicly available datasets
- A description of any restrictions on data availability
- For clinical datasets or third party data, please ensure that the statement adheres to our policy

The data used in this study are available in the FigShare database under accession code 10.6084/m9.figshare.24061197 [https://figshare.com/account/articles/24061197]. The data generated in this study are provided in the Source Data file. The Sequencing data generated in this study have been deposited in the BioProject database under the accession ID PRJNA991634. All material requests should be sent to Vincent J. Munster, vincent.munster@nih.gov.

## Research involving human participants, their data, or biological material

Policy information about studies with human participants or human data. See also policy information about sex, gender (identity/presentation), and sexual orientation and race, ethnicity and racism.

| | |
|---|---|
| Reporting on sex and gender | NA |
| Reporting on race, ethnicity, or other socially relevant groupings | NA |
| Population characteristics | NA |
| Recruitment | NA |
| Ethics oversight | NA |

Note that full information on the approval of the study protocol must also be provided in the manuscript.

# Field-specific reporting

Please select the one below that is the best fit for your research. If you are not sure, read the appropriate sections before making your selection.

[x] Life sciences   [ ] Behavioural & social sciences   [ ] Ecological, evolutionary & environmental sciences

For a reference copy of the document with all sections, see nature.com/documents/nr-reporting-summary-flat.pdf

# Life sciences study design

All studies must disclose on these points even when the disclosure is negative.

| | |
|---|---|
| Sample size | Challenge and transmission study: Power-analysis, animal group size determined to allow with 8 transmission pairs statistical significance with an alpha = 0.05 and power = 95%, if a difference of 75% in transmission was observed between the conditions. Experiments were performed with n=6 due to the public health emergency posed by the first emergence of Omicron. VOC comparison study: Data was collected in the framework of another study and sample size was based on power analysis: difference of >50% in virus neutralizing titers, with α=0.05 and power = 80%. |
| Data exclusions | Only sequencing data was included that passed quality control (e.g., subgenomic RNA copies >10/rxn). No additional data was excluded. |
| Replication | Animal study was done n=6 per group or n=3, representing biological replicates. For the in vitro assays n = 8, repeated twice. All replications were successful. |
| Randomization | Animals were randomly assigned to groups. Analysis were performed on data collected from these animals. Additional confounders such as sex were not controlled for. All animals had roughly the same age and weight. |
| Blinding | Data acquisition and analysis were blinded. |

# Reporting for specific materials, systems and methods

We require information from authors about some types of materials, experimental systems and methods used in many studies. Here, indicate whether each material, system or method listed is relevant to your study. If you are not sure if a list item applies to your research, read the appropriate section before selecting a response.

### Materials & experimental systems

| n/a | Involved in the study | |
|---|---|---|
| [ ] | [x] | Antibodies |
| [ ] | [x] | Eukaryotic cell lines |
| [x] | [ ] | Palaeontology and archaeology |
| [ ] | [x] | Animals and other organisms |
| [x] | [ ] | Clinical data |
| [x] | [ ] | Dual use research of concern |
| [x] | [ ] | Plants |

### Methods

| n/a | Involved in the study | |
|---|---|---|
| [x] | [ ] | ChIP-seq |
| [x] | [ ] | Flow cytometry |
| [x] | [ ] | MRI-based neuroimaging |

## Antibodies

| | |
|---|---|
| Antibodies used | 1. Secondary goat anti-hamster IgG Fc (Cat.No. 5220-0371 Lot. 10492253, Seracare; Reference number: 14-22-06 (https:// |

| | |
|---|---|
| | www.seracare.com/AntiHamster-IgG-HL-Antibody-PeroxidaseLabeled-5220-0371/)) spike-specific antibodies were used (diluted at 2500X)<br>2. Goat anti-hamster IgG cross-adsorbed secondary antibody (ThermoFisher, SA5-10284; Reference number AB_2868332 (https://www.thermofisher.com/antibody/product/Goat-anti-Syrian-Hamster-IgG-H-L-Cross-Adsorbed-Secondary-Antibody-Polyclonal/SA5-10284))<br>3. Using GenScript U864YFA140-4/CB2093 NP-1 (1:1000) specific anti-CoV<br>4. Vector Laboratories ImPress VR horse anti-rabbit IgG polymer, (https://vectorlabs.com/products/enzyme-polymer/immpress-vr-horse-anti-rabbit-igg-hrp-kit#documents))<br>5. Roche Tissue Diagnostics predilute (#790-4341, clone 2GV6)<br>6. Novus Biologicals at 1:500 (#NBP2-38790, polyclonal)<br>7. Vector Laboratories ImPress VR horse anti-rabbit IgG polymer (# MP-6401) |
| Validation | 1. Each lot is tested to assure specificity and lot-to-lot consistency using an in-house ELISA assay.<br>2. Each lot is tested to assure specificity and lot-to-lot consistency using an in-house assay.<br>3. Validation of cross-reactivity of SARS-CoV to SARS-CoV-2 in IHC was done in-house by embedding SARS-CoV-2 infected Vero cells in histogel and producing and staining histology slides.<br>4. This comes as a pre-dilute solution. It has been validated by Vector Laboratories to work appropriately on the Ventana Discovery ULTRA automated stainer. We run a Rabbit IgG control in place of primary antibody with known positive tissue for validation.<br>5. Validated against control tissues (e.g., spleen) by the manufacturer and in house. This is also provided as a pre-dilute antibody- we do no additional dilution. The concentration is at 0.4ug/mL<br>6. Validation by company by orthogonal strategies. The manufacturer compared IHC staining and corresponding PAX5 RNA-seq data for the same tissues.<br>7. This comes as a pre-dilute solution. It has been validated by Vector Laboratories to work appropriately on the Ventana Discovery ULTRA automated stainer. We run a Rabbit IgG control in place of primary antibody with known positive tissue for validation. |

## Eukaryotic cell lines

Policy information about cell lines and Sex and Gender in Research

| | |
|---|---|
| Cell line source(s) | VeroE6 UNC, provided by Ralph Baric (University of North Carolina, Chapel Hill, USA). Also available as VERO C1008 from ATCC (CRL-1586, https://www.atcc.org/products/all/crl-1586.aspx).<br>BHK, provided by Marshal Bloom (NIAID, Hamilton, MT, USA)). Also available from ATCC (CCL-10, https://www.atcc.org/products/ccl-10).<br>293T, provided by Sonja Best (NIAID, Hamilton, MT, USA). Also available from ATCC (CRL-3216 https://www.atcc.org/products/crl-3216). |
| Authentication | Cells were cytochrome B gene sequenced. |
| Mycoplasma contamination | Tested negative. |
| Commonly misidentified lines (See ICLAC register) | No commonly misidentified cell lines were used in the study. |

## Animals and other research organisms

Policy information about studies involving animals; ARRIVE guidelines recommended for reporting animal research, and Sex and Gender in Research

| | |
|---|---|
| Laboratory animals | Syrian Golden hamster, female and male, 4-6 weeks old, Hsd Han AURA |
| Wild animals | No wild animals were used in study. |
| Reporting on sex | Data was collected on male and female animals, but sex was not treated as a variable for the purpose and scope of this study. |
| Field-collected samples | No field collected samples were used in the study. |
| Ethics oversight | All animal experiments were conducted in an AAALAC International-accredited facility and were approved by the Rocky Mountain Laboratories Institutional Care and Use Committee following the guidelines put forth in the Guide for the Care and Use of Laboratory Animals 8th edition, the Animal Welfare Act, United States Department of Agriculture and the United States Public Health Service Policy on the Humane Care and Use of Laboratory Animals. Protocol number 2021-034-E and 2021-048-E. Work with infectious SARS-CoV-2 virus strains under BSL3 conditions was approved by the Institutional Biosafety Committee (IBC). For the removal of specimens from high containment areas, virus inactivation of all samples was performed according to IBC-approved standard operating procedures. |

Note that full information on the approval of the study protocol must also be provided in the manuscript.
